# Inhibition of CCCTC Binding Factor-Programmed Cell Death Ligand 1 Axis Suppresses Emergence of Chemoresistance Induced by Gastric Cancer-Derived Mesenchymal Stem Cells

**DOI:** 10.3389/fimmu.2022.884373

**Published:** 2022-04-27

**Authors:** Qianqian Wang, Chao Huang, Ying Ding, Shaodi Wen, Xin Wang, Shuwei Guo, Qiuzhi Gao, Zhihong Chen, Yuanyuan Zhao, Mei Wang, Bo Shen, Wei Zhu

**Affiliations:** ^1^ School of Medicine, Jiangsu University, Zhenjiang, China; ^2^ Department of Oncology, Jiangsu Cancer Hospital Affiliated to Nanjing Medical University, Nanjing, China; ^3^ Department of Gastrointestinal Surgery, Affiliated People’s Hospital of Jiangsu University, Zhenjiang, China

**Keywords:** mesenchymal stem cells, gastric cancer, 5-FU, paclitaxel, PD-L1, CTCF, chemotherapy resistance

## Abstract

**Background:**

Gastric cancer (GC) is the third leading cause of cancer-associated deaths worldwide. Stromal cells, especially mesenchymal stem cells (MSCs), play significant roles in the development of therapy resistance depending on their paracrine function. The PD-1/PD-L1 crosstalk between cancer and immune cells has been well studied. Emerging evidence suggests that PD-L1 also contributes to tumor resistance to therapy.

**Methods:**

Cell survival and apoptosis were assessed using CCK-8, colony formation, and flow cytometry assays. Protein alterations were analyzed *via* Western blot. Gene knockdown and overexpression were achieved with siRNA/shRNA and lentiviral infection, respectively. Drug effects on tumors *in vivo* were assessed with xenografts in nude mice. In addition, GC patient samples after chemotherapy treatment were collected to observe the relationship between chemotherapy effect and CTCF or PD-L1.

**Results:**

In response to 5-fluorouracil or paclitaxel treatment, GCMSC-CM enhanced the cell viability and decreased the apoptosis rate. Furthermore, blocking PD-L1 or CTCF in GC cells prevented GCMSC-induced drug resistance accompanied by a decline in cell stemness. Consistent with these *in vitro* observations, mice treated with GCMSC-CM showed a lower sensitivity to 5-fluorouracil. In addition, high expression of CTCF and PD-L1 was associated with poor chemotherapy progression in the clinic.

**Conclusion:**

Study results demonstrate a mechanism where GCMSC-CM promotes GC chemoresistance by upregulating CTCF-PD-L1 and provide strong evidence in support of targeting CTCF-PD-L1 signaling as a strategy to prevent resistance in the clinic.

## Introduction

Gastric cancer (GC) is the third leading cause of cancer-associated mortality worldwide due to the lack of effective therapies (1). Although the treatments have improved, the development of chemotherapy resistance is one of the most significant obstacles for effective cancer therapy (2). During conventional chemotherapy, such as treatment with 5-fluorouracil (5-FU), taxanes, and platinum, drug accumulation induces the expression of multi-drug resistance (MDR) genes in cancer cells. Therefore, the response to chemotherapy is often transient due to drug resistance (3). However, the underlying mechanisms of GC drug resistance are very complicated and still need more in-depth research. GC cells could bypass chemotherapy stress by expressing genes encoding efflux pumps, secreting exosomes, and altering some key signaling in favor of epithelial–mesenchymal transition (EMT), survival, and angiogenesis. Recent studies suggest that programmed death-ligand 1 (PD-L1) is involved in GC drug resistance (4).

PD-L1 is expressed in GC cells. Its expression in tumors assists in host immunity evasion and immune tolerance by reducing anti-tumor T-cell response (4). Immunotherapy based on this mechanism has been applied in the clinic. With further research, the drug resistance effect of PD-L1 will be gradually recognized (5, 6). Jiang et al. (7) have reported that PAR2 blockade reversed osimertinib resistance in lung cancer cells by attenuating PD-L1 expression and revealing communication between drug resistance and PD-1/PD-L1 signaling in lung cancer. However, there is a lack of beneficial strategies for targeting PD-L1 to overcome resistance to common chemotherapy regimens in GC. Indeed, our previous data indicated that GC-derived MSCs (GCMSC) induced CSC-like cells by enriching and targeting PD-L1 in GC cells (8).

Studies have suggested that the surrounding stromal cells in the tumor microenvironment, such as mesenchymal stem cells (MSCs), have an active role in tumor initiation, promotion, progression, and metastasis (9, 10). However, little is known about their potential influence on chemoresistance. Recently, tumor-associated MSCs were believed to modulate the chemotherapy response by either direct cell–cell interactions with tumor cells, or by the local release of soluble factors (11–16). For instance, MSCs induce resistance to chemotherapy through the release of platinum-induced fatty acids (13). In an orthotopic pancreatic tumor model, targeting tumor-initiating cells by nanovesicles derived from the MSC membranes and loaded with a CXCR3 antagonist enhanced the therapy outcome when administered in combination with gemcitabine (15). Research has shown that CD90^low^ MSCs activate EMT and resistance to temozolomide by increasing FOXS1 expression in glioma cells (16).

Previous studies from our group have focused on the promotion effect of MSCs on GC progression. It was demonstrated that bone marrow-derived MSCs promote GC cell proliferation and metastasis by upregulating c-Myc (17). GCMSCs were tightly correlated with PD-L1 expression in gastric tumor cells and tissues. However, little is known about whether GCMSC-CM can directly activate the intracellular signaling pathways *via* PD-L1 in GC cells, leading to tumor resistance to chemotherapeutic drugs. The current study sought to investigate whether GCMSCs are also involved in the development of resistance to 5-FU and paclitaxel (PTX) in GC patients. Our findings show that MSCs are potent mediators of resistance to 5-FU and PTX chemotherapy and reveal targets to enhance chemotherapy efficacy in GC patients. Finally, we provide evidence that CCCTC-binding factor (CTCF) and PD-L1 have an indispensable role in GCMSC-induced resistance to 5-FU and PTX in GC cells.

## Materials and Methods

### Cell Culture

Human GC cell lines (HGC-27, SGC-7901) were obtained from the Chinese Academy of Sciences Type Culture Collection Committee Cell Bank (Shanghai, China). HGC-27 and SGC-7901 cell lines were cultured in RPMI-1640 medium complemented with 10% fetal bovine serum (FBS, Gibco, USA) and maintained in 5% CO_2_ at 37°C in a humidified culture incubator. All reagents for cell culture were purchased from Invitrogen (Shanghai, China).

### GCMSC Isolation and Preparation of Conditioned Medium

GC patient samples were collected at the Jiangsu Cancer Hospital Affiliated with the Nanjing Medical University (Zhenjiang, Jiangsu, China) and the Affiliated People’s Hospital of Jiangsu University (Zhenjiang, Jiangsu, China). The collection of human tissues was approved by the Ethics Committee of Jiangsu University and written informed consent was obtained from each participant. As described previously, primary human GCMSCs were isolated from GC tissues.^17^ GC tissues were cut into 1-mm^3^ pieces and maintained in Dulbecco’s Modified Eagle’s Medium (DMEM; Gibco, USA) with 10% FBS at 37°C and 5% CO_2_ in a humidified culture incubator. After approximately 2 weeks, the fibroblast-like adherent cells identified as GCMSCs were trypsinized and passaged up to five times prior to use. GCMSCs were cultured in DMEM with 10% FBS in a humidified incubator containing 5% CO_2_ at 37°C. When the GCMSC confluence reached 80%, fresh culture medium was provided and collected after 48 h. The culture medium was then centrifuged at 2,000 *g* for 10 min to remove cellular debris. The supernatant was premixed with RPMI-1640 medium with 10% FBS at a volume ratio of 1:1 to generate the GCMSC conditioned medium (GCMSC-CM).

### Cell Viability Assay

Cell viability was detected using the Cell Counting Kit-8 assay (Beyotime, Jiangsu, China) following the manufacturer’s protocol. HGC-27 and SGC-7901 cells were seeded in 96-well plates at a density of 3×10^3^ cells/well and allowed to attach for 12 h. The medium was then replaced with fresh RPMI-1640 medium or GCMSC-CM with or without anti-PD-L1 and treated with different concentrations of 5-FU (Grand Pharma, China)/PTX (Beijing Union Pharm, China) in a total volume of 100 µl. Following culture for 24 h, 10 µl of CCK-8 reagent was added to 96-well plates and cultured for a further 1 h. Cell viability was determined by measuring optical density at 450 nm with a multiwell plate reader (FLx800, BioTek, Winooski, VT, USA).

### Western Blot Analysis

Cells or tissues were lysed with RIPA buffer (Invitrogen, Carlsbad, CA, USA) according to the standard protocols. Equal amounts of protein lysates were separated by 12% sodium dodecyl sulfate-polyacrylamide gel electrophoresis and blotted onto polyvinylidene fluoride membranes (Millipore, Billerica, MA, USA). The membranes were blocked with 5% non-fat milk and incubated with primary antibodies against GAPDH, PD-L1, MDR1, CD44, OCT4, and CTCF (all diluted to 1:1,000, Cell Signaling Technology, USA) overnight at 4°C, followed by incubation with goat anti-mouse and anti-rabbit secondary antibodies (all diluted to 1:3,000, Cell Signaling Technology, USA) conjugated with horseradish peroxidase for 1 h at 37°C. Signals were detected using ECL reagents (Millipore, Billerica, MA, USA).

### Annexin V/PI Apoptosis Analysis

Cells in six-well plates were incubated with or without GCMSC-CM and anti-PD-L1 for 24 h and then treated with or without 5-FU or PTX for 24 h before the cells were trypsinized, washed with PBS, and resuspended. Apoptosis was detected using the annexin V/PI apoptosis kit (BD Biosciences, San Jose, CA, USA) according to the manufacturer’s instructions. The stained cells were analyzed by flow cytometry (FACSCalibur, BD Biosciences) with FlowJo software.

### siRNA/shRNA and Lentivirus Transduction

Specific shRNA against PD-L1 (5’-GCACATCCTCCAAATGAAAGG-3’) and its corresponding NC (shNC) were acquired from GenePharma Technologies (Shanghai, China). SGC-7901 cells were transduced with shPD-L1 to generate SGC-7901 cell lines expressing shPD-L1. Then, SGC-7901 cells were treated with appropriate concentrations of GCMSC-CM, 5-FU/PTX, or their combination for an additional 24 h. To establish stable PD-L1-overexpressing GC cells, HGC-27 cells were transfected with a recombinant lentivirus (pLV-PD-L1-puro-GFP) and selected with puromycin (2 μg/ml) for 2 weeks. The transfection procedure was carried out strictly according to the manufacturer’s instructions (GenePharma Technologies, China). The siRNA targeting human CTCF (5’-GUGCAAUUGAGAACAUUAUTT-3’) and non-targeting control siRNA (siNC) were obtained from GenePharma Technologies (Shanghai, China). HGC-27 cells were transiently transfected with siNC and siCTCF with Lipofectamine 2000 (Thermo Fisher Scientific, USA) in serum-free medium for 6 h when cell confluence reached 60%, followed by recovery with medium complemented with 10% FBS or GCMSC-CM. At 24 h post-transfection, the cells were stimulated with the indicated concentrations of 5-FU. After 24 h, the cells were collected for use in further experiments.

### Colony-Forming Assay

HGC-27 and SGC-7901 cells were cultured with GCMSC-CM, anti-PD-L1 alone, or a combination of both for 24 h and then treated with 5-FU/PTX. Cells were digested with 0.05% trypsin after 24 h, and 500 single cells were seeded in a 6-cm well plate in triplicate. After about 10 days, cells were fixed with 4% paraformaldehyde and stained with 0.5% crystal violet solution (Sigma, USA) for 30 min. Colonies containing more than 50 cells were counted and photographed.

### *In Vivo* Experiment

First, 4- to 5-week-old male BALB/c nude mice were obtained from the Model Animal Research Center of Nanjing University (Nanjing, China) and maintained in the animal facility at Jiangsu University. All animal protocols were approved by the local ethics committee of Jiangsu University (Jiangsu, China). Next, approximately 1×10^6^ SGC-7901 cells or SGC-7901 cells expressing shPD-L1 were subcutaneously injected into the right flanks of nude mice and the animals were monitored regularly. The tumor volume was calculated every 3 days using calipers and the following formula: tumor volume = length × width^2^/2. When the xenograft tumor volume reached 100 mm^3^ (about 9 days), the mice were divided into five groups: (1) mice injected with shNC SGC-7901 cells; (2) mice injected with shNC SGC-7901 cells and treated with 5-FU; (3) mice injected with shNC SGC-7901 cells and treated with GCMSC-CM and 5-FU; (4) mice injected with shPD-L1 SGC-7901 cells and treated with 5-FU; and (5) mice injected with shPD-L1 SGC-7901 cells and treated with GCMSC-CM and 5-FU. Each group had five mice. Mice in groups 4 and 5 received 5-FU (20 mg/kg) *via* an intraperitoneal injection every 3 days for 21 days. A peritumoral injection schedule with 200 µl of GCMSC-CM for 21 successive days was performed in groups 3 and 5 every 2 days. After 21 days, the mice were sacrificed and the tumors were harvested and weighed for use in further analyses.

### Immunohistochemistry

Samples from 19 GC patients after chemotherapy treatment were obtained through the Jiangsu Cancer Hospital (Nanjing, Jiangsu). All studies were conducted in accordance with the Ethics Committee of Jiangsu Cancer Hospital and written informed consent was obtained from each participant. GC tissues from mice or patients were embedded in paraffin and sectioned. For the histological study, tumor sections were stained with anti-PD-L1, anti-MDR1, anti-CD44, anti-CTCF, and anti-Ki67 (all diluted to 1:100, Cell Signaling Technology, USA) overnight at 4°C and imaged with an AxioLab microscope equipped with an AxioCam HRC CCD camera (Zeiss, Germany).

### Statistical Analysis

Statistical analyses were performed using GraphPad Prism 7.0. Statistical comparisons between two groups were performed using the Student’s *t*-test. Multiple comparisons among groups were performed using one-way analysis of variance. The Kruskal–Wallis *H* test was used to analyze the differences between *in vivo* tumor growths. *p* < 0.05 was considered to be statistically significant.

## Results

### GCMSC-CM Enhances the Resistance of GC cells to 5-FU and PTX and Blocking PD-L1 Weakens These Effects

**Figure 1:**
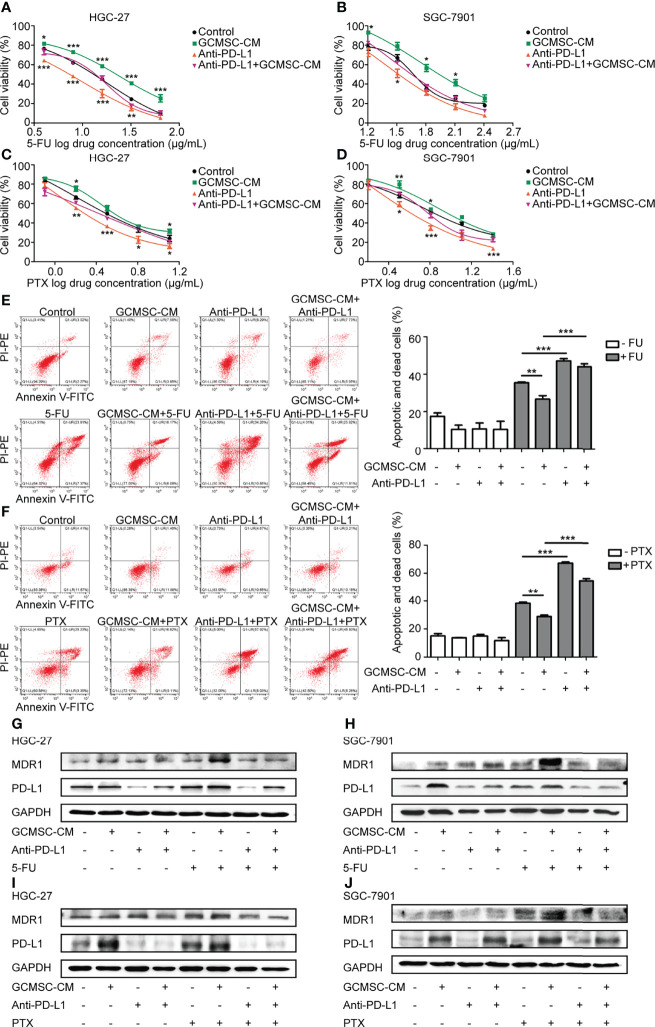
GCMSC-CM enhances GC cell resistance to 5-FU and PTX, while PD-L1 blocking weakens these effects. **(A–D)** HGC-27 and SGC-7901 cells co-cultured with GCMSC-CM and anti-PD-L1 were treated with different concentrations of 5-FU and PTX to detect cell viability using CCK-8 assay. **(E, F)** Annexin V-FITC/PI flow cytometry assay was used to analyze the apoptosis rate of HGC-27 cells. Panels on the right are quantified images. **(G–J)** PD-L1 and MDR1 levels in HGC-27 and SGC-7901 cells were examined by Western blot. *p < 0.05, **p < 0.01, ***p < 0.001. Concentration of PD-L1 neutralizing antibody was 2 μg/mL (eBioscience).

To evaluate the role of GCMSC-CM in the chemotherapeutic effect in GC microenvironment, HGC-27 or SGC-7901 cells were exposed to different concentrations of 5-FU or PTX (**Supporting Figure 1**) with GCMSC-CM. Cell viability was analyzed using a CCK-8. GCMSC-CM led to a higher cell survival rate after the 5-FU or PTX treatment, suggesting that GCMSC-CM protects GC cells from being killed by chemotherapeutics (**Figures 1A**–**D**). To elucidate the importance of PD-L1 in GCMSC-CM-induced chemoresistance, the PD-L1 expression was blocked using the PD-L1 neutralizing antibody (anti-PD-L1). The changes caused by GCMSC-CM were reversed by depleting PD-L1 in HGC-27 and SGC-7901 cells. Next, annexin V-FITC/PI flow cytometry assay was performed to detect cell apoptosis. The 5-FU and PTX induced the basal cell apoptosis in HGC-27 cells. Flow cytometry data indicated that GCMSC-CM significantly reduced the apoptosis rate induced by 5-FU and PTX, while blocking the PD-L1 expression in GC cells increased the apoptosis rate (**Figures 1E, F**). The expression of PD-L1 and MDR1 in GC cells was further detected using Western blotting. An obvious positive correlation was observed between PD-L1 and MDR1 (**Figures 1G**–**J**).

### GCMSC-CM Increases GC Cell Stemness by Upregulating PD-L1 and Leads to Chemotherapy Resistance

**Figure 2:**
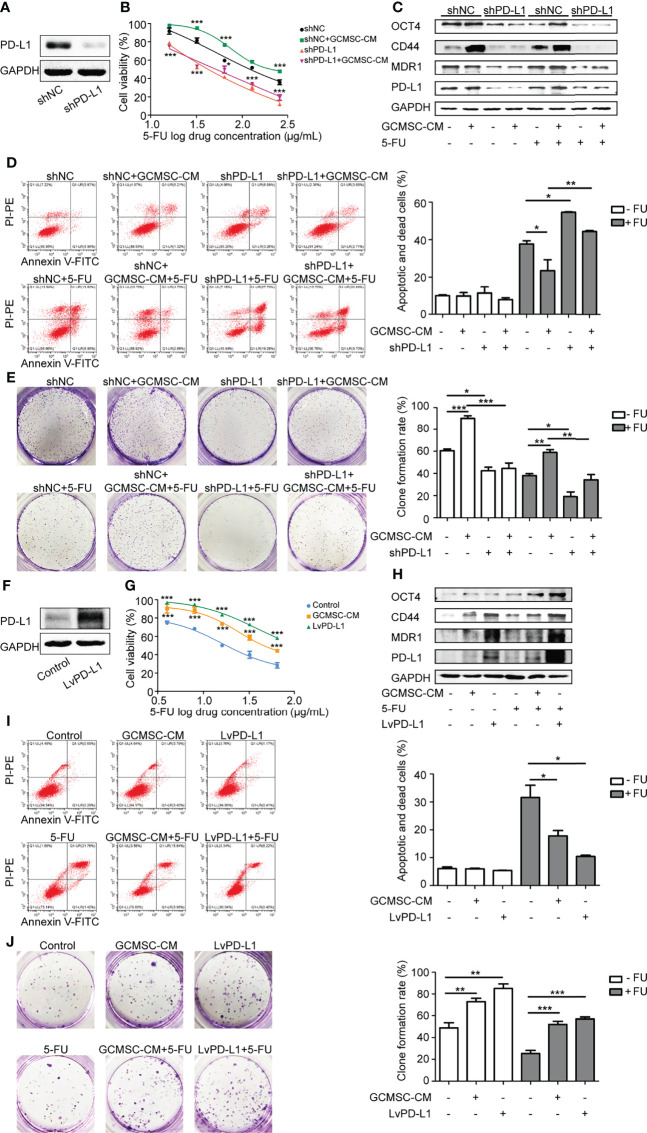
GCMSC-CM increases GC cell stemness by upregulating PD-L1 and leads to chemotherapy resistance. **(A)** Transfection efficiency of shPD-L1 was tested by Western blot. CCK-8 **(B)**, Western blot **(C)**, flow cytometry **(D)**, and colony-forming assay **(E)** were performed in shPD-L1 SGC-7901 cells following GCMSC-CM and 5-FU treatment. Panels on the right are quantified images. **(F)** PD-L1 level in LvPD-L1 HGC-27 cells was tested by Western blot. CCK-8 **(G)**, Western blot **(H)**, flow cytometry **(I)**, and colony-forming assay **(J)** were performed in LvPD-L1 HGC-27 cells following GCMSC-CM and 5-FU treatment. Panels on the right are quantified images. *p < 0.05, **p < 0.01, ***p < 0.001.

Based on the aforementioned results, it was hypothesized that GCMSC-CM enhances resistance to chemotherapy by upregulating PD-L1 in GC cells. To further verify this hypothesis, the expression of PD-L1 in SGC-7901 cells was knocked down and the transfection efficiency was detected (**Figure 2A**). Compared to the shNC+GCMSC-CM group, cells in the shPD-L1+GCMSC-CM group showed a significantly lower cell viability and MDR1 expression and a higher apoptosis rate (*p* < 0.05) (**Figures 2B**–**D**). Since PD-L1 is known as a critical protein for maintaining tumor cell stemness, it was also demonstrated that co-culture with GCMSC-CM led to an increased colony formation ability. These effects were reversed in shPD-L1 SGC-7901 cells (**Figure 2E**). Moreover, basal expression of CD44 and OCT4 in the GCMSC-CM group showed no significant change without the 5-FU treatment. An obvious upregulation was observed following the 5-FU treatment, which was decreased by the PD-L1 knockdown. The PD-L1 expression was increased in order to confirm its effects on HGC-27 cell resistance to 5-FU. The high expression of PD-L1 in LvPD-L1 HGC-27 cells is verified in **Figure 2F**. PD-L1 overexpression significantly increased cell viability, colony formation capacity, and protein levels of PD-L1, MDR1, CD44, and OCT4 (**Figures 2G**–**J**). In addition, these results were also independently validated using the PTX treatment (**Supporting Figure 2**). This might explain the results in which GCMSC-CM-treated GC cells showed a higher resistance to 5-FU by upregulating PD-L1 and stemness.

### CTCF Is Essential for Maintenance of Cell Stemness and High Expression of PD-L1 Caused by GCMSC-CM

**Figure 3:**
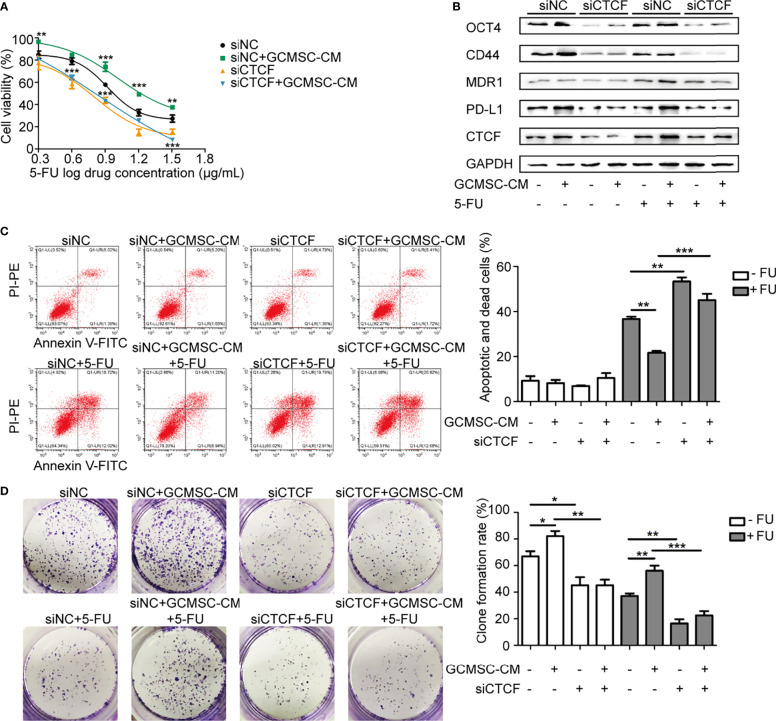
CTCF is essential for maintenance of cell stemness and high expression of PD-L1 caused by GCMSC-CM. CCK-8 **(A)**, Western blot **(B)**, flow cytometry **(C)**, and colony-forming assay **(D)** were performed in siCTCF HGC-27 cells following GCMSC-CM and 5-FU treatment. Panels on the right are quantified images. *p < 0.05, **p < 0.01, ***p < 0.001.

Our previous studies have demonstrated that PD-L1 interacts with CTCF. Considering that CTCF is a stemness-related protein, it was hypothesized that GCMSC-CM may hinder the 5-FU therapeutic effect by regulating CTCF, leading to high expression of PD-L1. The loss of CTCF dramatically decreased the survival of HGC-27 cells after exposure to 5-FU (**Figures 3A**–**D**). In comparison to the siNC+GCMSC-CM cells, the siCTCF+GCMSC-CM group cells became significantly more sensitive to 5-FU. Consistently, the CTCF inhibition was accompanied by a lower clone formation number and lower expression of PD-L1, MDR1, CD44, and OCT4, indicating that GCMSC-CM promoted GC cell resistance to 5-FU. These effects were partly dependent on CTCF-PD-L1.

### GCMSC-CM Reduces Mouse Sensitivity to 5-FU and PD-L1 Knockdown Recovers It

**Figure 4:**
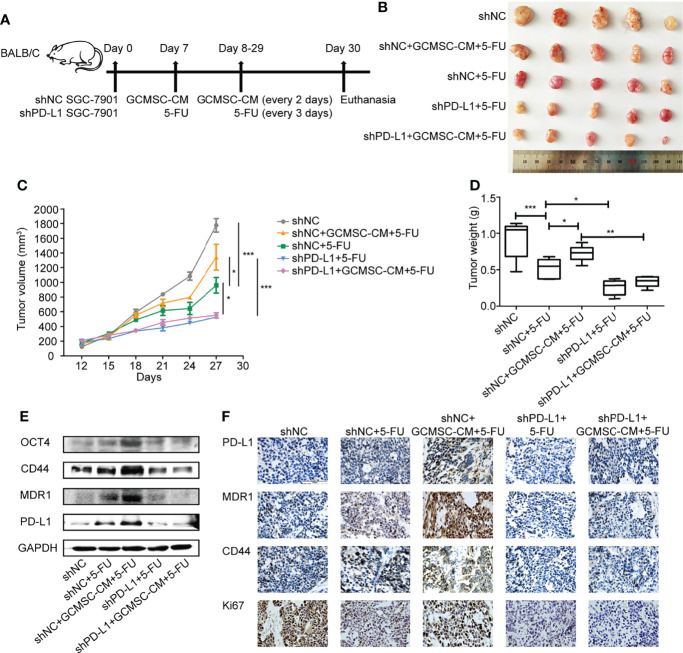
GCMSC-CM reduces sensitivity of mice to 5-FU and PD-L1 knockdown recovers it. **(A)** Schematic representation of *in vivo* experiment. **(B)** Representative images of tumors from the indicated group. **(C)** Tumor volumes of SGC-7901 xenografts in mice. **(D)** Comparison of tumor weights in each group on day 30. **(E)** Expression of PD-L1, MDR1, CD44, and OCT4 in SGC-7901 xenograft tissues from different treatment groups was detected *via* Western blot. **(F)** Expression of PD-L1, MDR1, CD44, and Ki67 in SGC-7901 xenograft tissues was detected by immunohistochemistry (scale bar: 100 μm). *p < 0.05, **p < 0.01, ***p < 0.001.

Based on the promising *in vitro* data, we next examined whether GCMSC-CM-induced drug resistance was altered *in vivo* following 5-FU therapy. **Figure 4A** shows the schematic diagram of the *in vivo* experiment. Single 5-FU markedly altered tumor progression in mouse models, and GCMSC-CM treatment weakened the ability of 5-FU to restrain tumor progression. After the 5-FU therapy, GCMSC-CM somewhat promoted gastric tumor growth. However, mouse injections with shPD-L1 SGC-7901 cells significantly inhibited tumor growth (**Figures 4B**–**D**). This result was correlated with the effect on the expression of PD-L1, MDR1, CD44, and OCT4 in mouse tissues (**Figure 4E**). The expression of Ki67, PD-L1, MDR1, and CD44 was also confirmed *via* immunohistochemical staining (**Figure 4F**).

### PD-L1, CTCF, and Patient Survival

**Figure 5:**
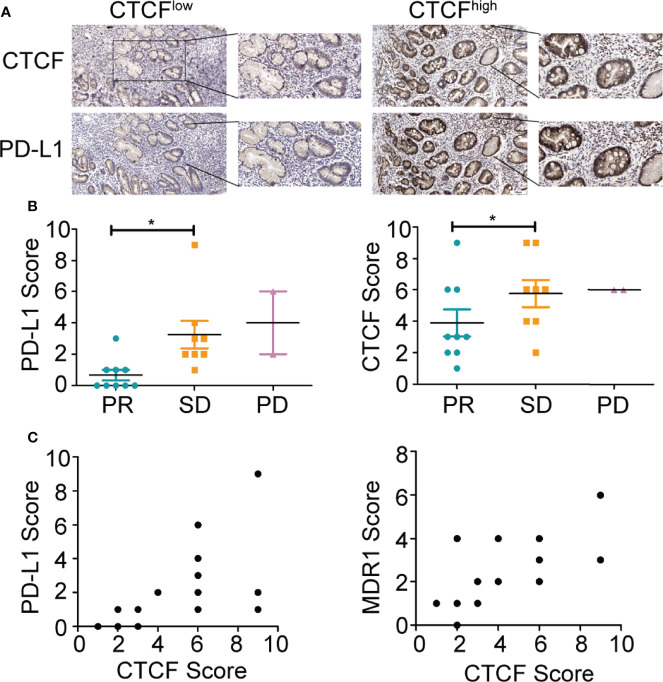
PD-L1 and CTCF are positively correlated in clinical GC samples. **(A)** Representative immunohistochemical staining images of CTCF and PD-L1 in GC tissues from patients (scale bar: 100 μm, 40 μm). **(B)** Relationship between CTCF or PD-L1 and chemotherapy effect. **(C)** Relationship between CTCF and PD-L1 or MDR1 in GC tissues from patients. *p < 0.05.

Furthermore, the expression of CTCF and PD-L1 in 19 clinical specimens was evaluated following independent immunohistochemistry analysis (**Figure 5A**). The staining intensity was scored as 0 (negative), 1 (weak), 2 (medium), or 3 (strong), whereas the staining extent was scored as 0 (0% of the staining area), 1 (1%–25%), 2 (26%–50%), 3 (51%–75%), and 4 (76%–100%). The final scores of the targeted protein expression were calculated using the products of the staining intensity and the extent scores. As expected, high expression of CTCF and PD-L1 was associated with poor chemotherapy progression (**Figure 5B**). However, a positive relationship between CTCF and PD-L1 or MDR1 was not observed across a number of tumor tissues in GC patients (**Figure 5C**), which was likely due to the insufficient number of tissue samples.

## Discussion

Worldwide, advanced GC exhibits poor prognosis with surgery alone. Thus, surgery combined with chemotherapy and immunotherapy have become very important (18). Chemotherapy, including 5-FU, adriamycin, PTX, and platinum drugs, remains one of the fundamental methods of GC treatment and has efficiently improved patient prognosis. Drug resistance is one of the biggest challenges for 5-FU and PTX treatments in GC patients, directly leading to clinical drug therapy failure (19). In the history of GC chemotherapy, oral anti-metabolite 5-FU, as well as trastuzumab (HER2 neutralizing antibody) and platinum preparations (cisplatin-CDDP) have been adopted as first-line chemotherapy regimens for unresectable and/or recurrent GC patients (20). Second- and third-line chemotherapy regimens consist of taxanes, ramucirumab, and nivolumab. Therefore, 5-FU and PTX, which are critical in the clinic in the present study, were selected. The 5-FU inhibits the DNA synthesis by reducing thymidylate synthase activity, while PTX inhibits depolymerization of tubulin and subsequent cell division (21, 22). Dysregulation of various components in distinct pathways is the major common cause for drug resistance, including alterations in drug transport, EMT transition, miRNA dysregulations, cell cycle changes, autophagy regulation, DNA damage repair machinery, tumor microenvironment interactions, and stemness enhancement (23).

Recently, increasing evidence has indicated that MSCs become activated during drug treatment and secrete factors that protect tumor cells against a range of chemotherapeutics (24, 25). MSCs derived from adipose tissues are involved in a gradual loss of the repair capacity of oxidative DNA damage and accumulation of DNA damage, which are associated with drug resistance (26). There is also enough evidence to demonstrate that MSCs increase SNHG7 expression in pancreatic cancer cells, followed by promoting the folfirinox resistance (27). However, there are rare reports about the role of GCMSCs in 5-FU and PTX resistance in GC. The present study showed that GCMSC-CM enhanced cell viability and decreased cell apoptosis rate when GC cells were treated with 5-FU and PTX.

Our previous studies have demonstrated that GCMSC-derived IL-8 induces PD-L1 expression in GC cells *via* the STAT3/mTOR-c-Myc signaling axis and that blocking IL-8 and IL-15 derived from GCMSCs overcomes the immune escape induced by PD-L1 in GC cells (8). The PD-L1 function in this process was also explored. Notably, the results showed that PD-L1 and MDR1 expression levels were elevated in GC cells compared to the parallel cells, indicating PD-L1 participation in generation of drug resistance. Furthermore, a neutralizing antibody was used to evaluate the effect of PD-L1 in GCMSC-CM-induced drug resistance. In recent years, it has been established that PD-L1 is associated with radiotherapy and chemotherapy resistance in various cancers (28, 29). The PD-L1 expression was considered to be correlated with cisplatin and trastuzumab resistance in cancer cells (30, 31). In accordance with previous reports (32), the PD-L1 knockdown did not only further suppress the GC cell proliferation and recover cell apoptosis, but also significantly suppressed the colony-forming ability and MDR1 protein levels. The study of PD-1/PD-L1 crosstalk between cancer and immune cells suggested that PD-L1 may protect GC cells from 5-FU and PTX other than regulate immune tolerance. To further explore this mechanism, it was hypothesized that CTCF participates in the process based on our previous studies. Recently, studies have shown that CTCF, which is a zinc finger protein, promotes GC progression *via* the LINC01207 and Wnt signaling pathway (33, 34). Expectedly, the inhibition of CTCF attenuated cell viability accompanied by low expression of PD-L1 and promoted cell apoptosis in GC cells treated with 5-FU, revealing the reversal effect of targeting CTCF in GC resistant to 5-FU. Thus, we speculated that targeting CTCF-PD-L1 could overcome 5-FU resistance in GC by increasing cancer cell stemness and downstream signaling. To validate the *in vitro* results, a xenograft tumor model was constructed to confirm that GCMSC-CM weakened the effect of 5-FU in mice by upregulating PD-L1 *in vivo.*

Furthermore, CTCF and PD-L1 expression was a negative prognostic factor for GC patients after treatment with chemotherapeutic drugs. Unfortunately, a further clinical investigation is still necessary because the number of patients with available tissue for the IHC testing was insufficient for firm conclusions. Taken together, the present study results for the first time demonstrated that GCMSC promoted resistance to 5-FU by regulating the CTCF-PD-L1 axis. These studies have the potential to provide fundamental knowledge to increase our understanding of drug resistance that potentiates anti-tumor effects of 5-FU and to improve the patient’s overall survival in the near future.

## Conclusions

The resistance observed in GC cells was enhanced by GCMSC-CM and reversed by CTCF-PD-L1 inhibition. Further investigation of this therapeutic strategy could improve its therapeutic efficacy in the clinic.

## Data Availability Statement

The raw data supporting the conclusions of this article will be made available by the authors, without undue reservation.

## Ethics Statement

The studies involving human participants were reviewed and approved by the local ethics committee of Jiangsu University. The patients/participants provided their written informed consent to participate in this study. The animal study was reviewed and approved by the local ethics committee of Jiangsu University.

## Author Contributions

QW: Experiment design and completion, data acquisition and analysis, software, writing—original draft preparation, and editing. YD, CH, SW, XW, SG, QG, and ZC: Writing—original draft preparation and editing. YZ, MW, BS, and WZ: Writing—reviewing, and supervision. All authors contributed to the article and approved the submitted version.

## Funding

This study was supported by the National Science Foundation of China (Grant Nos. 81972822 and 81972313).

## Conflict of Interest

The authors declare that the research was conducted in the absence of any commercial or financial relationships that could be construed as a potential conflict of interest.

## Publisher’s Note

All claims expressed in this article are solely those of the authors and do not necessarily represent those of their affiliated organizations, or those of the publisher, the editors and the reviewers. Any product that may be evaluated in this article, or claim that may be made by its manufacturer, is not guaranteed or endorsed by the publisher.

## Supplementary Material

Supplementary Figure 1 GC cell IC50 to 5-FU and PTX. HGC-27 and SGC-7901 cells were treated with different concentrations of 5-FU or PTX to detect cell viability *via* CCK-8 assay. **(A)** IC50 of HGC-27 cells to 5-FU was 15.4 µg/mL. **(B)** IC50 of HGC-27 cells to PTX was 3.16 µg/mL. **(C)** IC50 of SGC-7901 cells to 5-FU was 57.13 µg/mL. **(D)** IC50 of SGC-7901 cells to PTX was 6.28 µg/mL.

Supplementary Figure 2 GCMSC-CM increases GC cell stemness by up-regulating PD-L1 and leads to chemotherapy resistance to PTX. CCK-8 **(A)**, flow cytometry **(B)**, and colony forming assay **(C)** were performed in PD-L1-knockdown SGC-7901 cells following GCMSC-CM and PTX treatment. Panels on the right are quantified images. **(D)** Expression of PD-L1, MDR1, CD44, and OCT4 in SGC-7901 cells was detected by western blot. CCK-8 **(E)**, flow cytometry **(F)**, and colony forming assay **(G)** were performed in Lv-PD-L1 HGC-27 cells following GCMSC-CM and PTX treatment. Panels on the right are quantified images. (H) Expression of PD-L1, MDR1, CD44, and OCT4 in HGC-27 cells was detected by western blot. **P*<0.05, ***P*<0.01, ****P*<0.001.
