# Supplementary figures and images for: Inhibition of CCCTC Binding Factor-Programmed Cell Death Ligand 1 Axis Suppresses Emergence of Chemoresistance Induced by Gastric Cancer-Derived Mesenchymal Stem Cells
Source: Front Immunol. 2022 Apr 27;13:884373. doi: 10.3389/fimmu.2022.884373 (PMC9095388; doi:10.3389/fimmu.2022.884373)

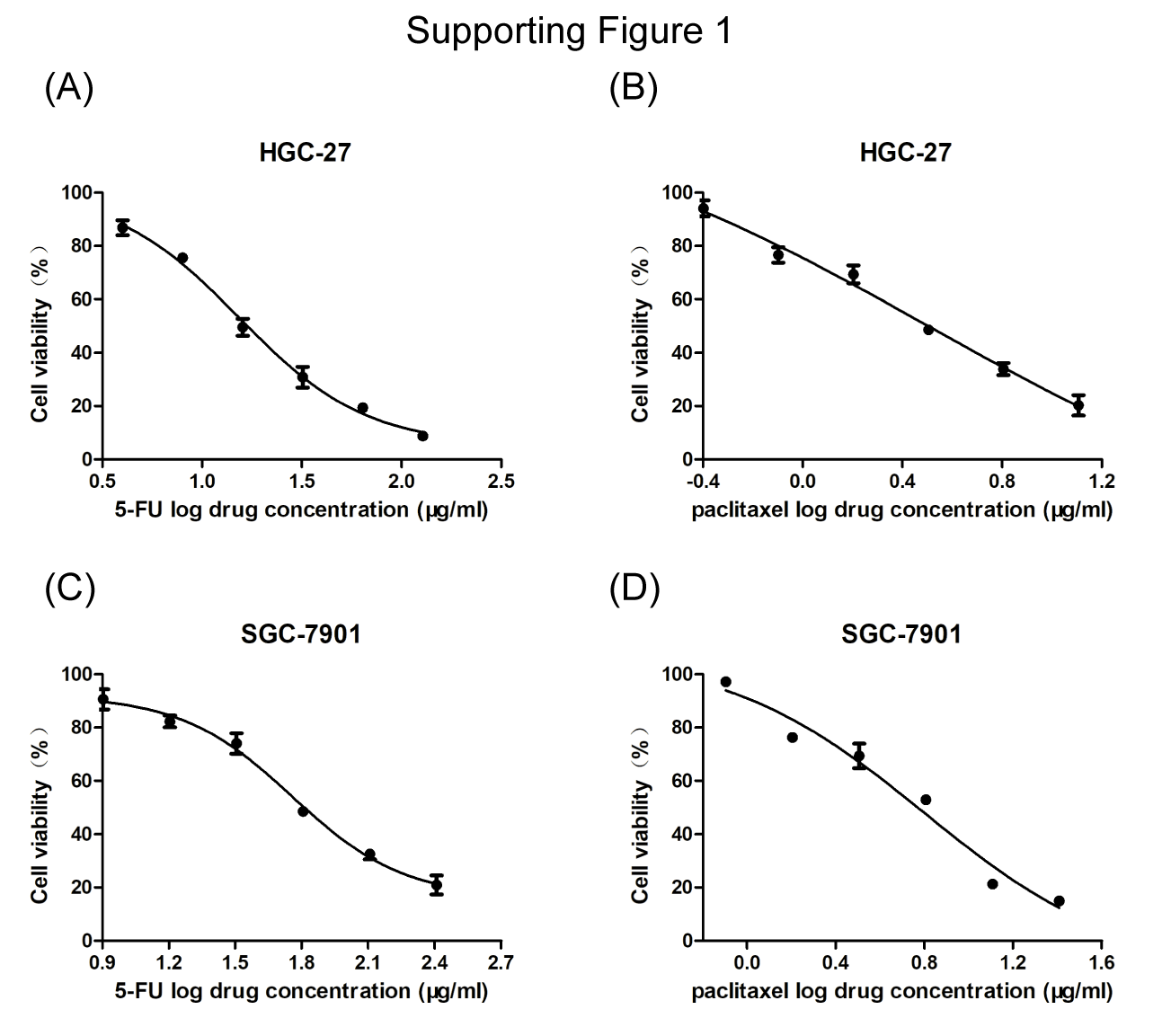

Supplement: Supplementary Figure 1 — GC cell IC50 to 5-FU and PTX. HGC-27 and SGC-7901 cells were treated with different concentrations of 5-FU or PTX to detect cell viability via CCK-8 assay. (A) IC50 of HGC-27 cells to 5-FU was 15.4 µg/mL. (B) IC50 of HGC-27 cells to PTX was 3.16 µg/mL. (C) IC50 of SGC-7901 cells to 5-FU was 57.13 µg/mL. (D) IC50 of SGC-7901 cells to PTX was 6.28 µg/mL. [file Image_1.tif]

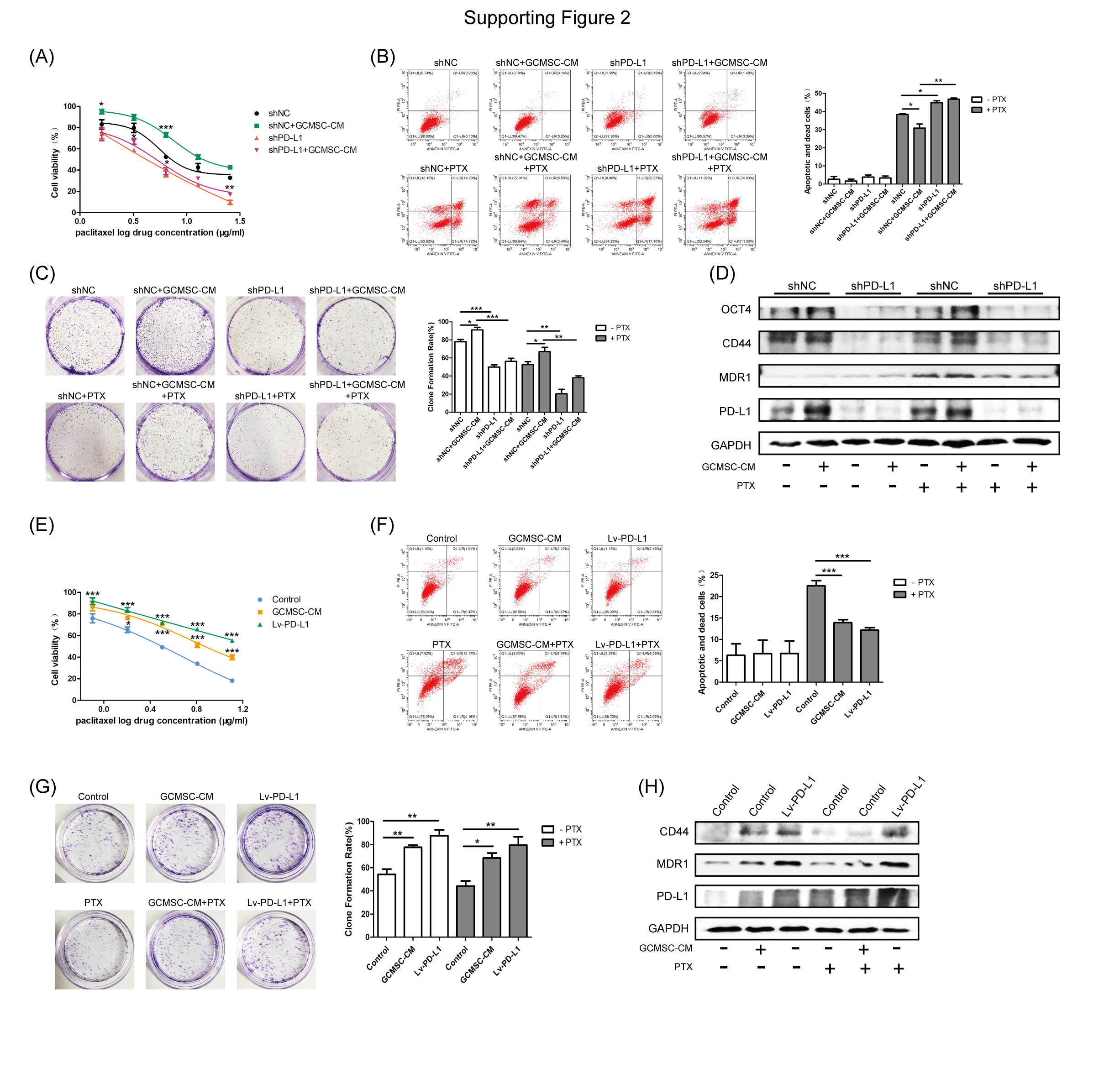

Supplement: Supplementary Figure 2 — GCMSC-CM increases GC cell stemness by up-regulating PD-L1 and leads to chemotherapy resistance to PTX. CCK-8 (A), flow cytometry (B), and colony forming assay (C) were performed in PD-L1-knockdown SGC-7901 cells following GCMSC-CM and PTX treatment. Panels on the right are quantified images. (D) Expression of PD-L1, MDR1, CD44, and OCT4 in SGC-7901 cells was detected by western blot. CCK-8 (E), flow cytometry (F), and colony forming assay (G) were performed in Lv-PD-L1 HGC-27 cells following GCMSC-CM and PTX treatment. Panels on the right are quantified images. (H) Expression of PD-L1, MDR1, CD44, and OCT4 in HGC-27 cells was detected by western blot. *P<0.05, **P<0.01, ***P<0.001. [file Image_2.tif]
